# Iron-Binding Protein Degradation by Cysteine Proteases of *Naegleria fowleri*

**DOI:** 10.1155/2015/416712

**Published:** 2015-05-18

**Authors:** Moisés Martínez-Castillo, Gerardo Ramírez-Rico, Jesús Serrano-Luna, Mineko Shibayama

**Affiliations:** ^1^Department of Infectomics and Molecular Pathogenesis, Center for Research and Advanced Studies of the National Polytechnic Institute, Avenida IPN 2508, 07360 Mexico City, Mexico; ^2^Department of Cell Biology, Center for Research and Advanced Studies of the National Polytechnic Institute, Avenida IPN 2508, 07360 Mexico City, Mexico; ^3^Faculty of Professional Studies, Autonomous University of Mexico, Campus Cuautitlán, Km 2.5 Carretera Cuautitlán-Teoloyucan, 54714 Cuautitlán Izcalli, Mexico

## Abstract

*Naegleria fowleri* causes acute and fulminant primary amoebic meningoencephalitis. This microorganism invades its host by penetrating the olfactory mucosa and then traveling up the mesaxonal spaces and crossing the cribriform plate; finally, the trophozoites invade the olfactory bulbs. During its invasion, the protozoan obtains nutrients such as proteins, lipids, carbohydrates, and cationic ions (e.g., iron, calcium, and sodium) from the host. However, the mechanism by which these ions are obtained, particularly iron, is poorly understood. In the present study, we evaluated the ability of *N. fowleri* to degrade iron-binding proteins, including hololactoferrin, transferrin, ferritin, and hemoglobin. Zymography assays were performed for each substrate under physiological conditions (pH 7 at 37°C) employing conditioned medium (CM) and total crude extracts (TCEs) of *N. fowleri*. Different degradation patterns with CM were observed for hololactoferrin, transferrin, and hemoglobin; however, CM did not cause ferritin degradation. In contrast, the TCEs degraded only hololactoferrin and transferrin. Inhibition assays revealed that cysteine proteases were involved in this process. Based on these results, we suggest that CM and TCEs of *N. fowleri* degrade iron-binding proteins by employing cysteine proteases, which enables the parasite to obtain iron to survive while invading the central nervous system.

## 1. Introduction

*Naegleria fowleri* is a free-living amoeba that causes primary amoebic meningoencephalitis (PAM) in humans. This protozoan gains access to the central nervous system (CNS) by penetrating the olfactory neuroepithelium and migrating through olfactory nerves and reaching the olfactory bulbs [1–5]. Immunohistochemical studies of the early events of infection in a murine model have shown that the amoebae induce intense mucus secretion and an inflammatory reaction in the nasal cavity [6]. At later stages of infection, tissue damage characterized by extensive lytic and necrotic areas, hemorrhage, and cellular debris has been reported. In hemorrhagic areas, the amoebae have been observed to carry several ingested erythrocytes [7]. The molecular mechanisms employed by* N. fowleri* to process and degrade cells or molecules are poorly studied. However, it is known that, during invasion,* N. fowleri* is able to release proteolytic proteins including naegleriapores A and B, phospholipases, glycosidases, neuraminidase, elastase, and other proteases, such as cathepsin B and mucinases [8–14]. Some of these proteases have been evaluated using* in vitro* systems. These studies evaluated specific human substrates such as IgA, IgG, IgM, collagen, fibronectin, hemoglobin, albumin, mucus, and elastin [2, 12, 13]. Other investigations have analyzed proteins associated with iron. In invasive pathogens such as bacteria, the acquisition of iron is crucial to division and survival; additionally, previous studies have shown that bacteria can acquire iron from a substantial number of iron-binding proteins, including transferrin, lactoferrin, hemopexin, ferritin, hemoglobin, the hemoglobin/haptoglobin complex, and human serum albumin [15–17]. In some protozoans, such as* Tritrichomonas foetus*,* Trichomonas vaginalis*,* Toxoplasma gondii,* and* Entamoeba histolytica*, the expression of lactoferrin-binding proteins has been described, and these parasites use hololactoferrin as an iron source for* in vitro* growth [18]. Another mechanism involved in the acquisition of iron from lactoferrin has been reported in promastigotes of* Leishmania chagasi*, which use a surface reductase that recognizes and reduces ferric iron to the accessible ferrous form (Fe^2+^) [19]. Cysteine proteases that cleave lactoferrin have also been reported in* E. histolytica* [20]. In contrast, there is less information regarding iron acquisition in the* Naegleria* genus; consequently, we analyzed the ability of* N. fowleri* to degrade molecules associated with iron. We determined that the proteases released from free-living amoebae were able to degrade iron-binding human proteins, including hololactoferrin, hemoglobin, and holotransferrin, but not ferritin. It is possible that the degradation of iron-binding proteins could play a role in PAM progression in both human and animal models of the disease.

## 2. Materials and Methods

### 2.1. Amoebic Cultures

The pathogenic strain* N. fowleri* (ATCC 30808) was used in all experiments. However, to maintain the amoebic virulence, trophozoites were instilled in mice; seven days later, the brains were recovered in Bacto Casitone medium with antibiotics. Finally, the culture was maintained in axenic conditions in 2% (w/v) Bacto Casitone medium supplemented with 10% (v/v) fetal bovine serum (FBS; Equitech-Bio, USA) at 37°C. The trophozoites were harvested during the exponential growth phase (48 h).

### 2.2. Sample Preparation

Total crude extracts (TCEs) were obtained as previously described with some modifications [21]. Briefly,* N. fowleri* trophozoites were removed from the culture flask surface by chilling in an ice bath for 20 min, centrifuged at 800 ×g for 10 min, and washed with phosphate buffered saline (PBS) (pH 7.2). Subsequently, the trophozoite pellets were incubated at 37°C for 30 min and then disrupted by five freeze-thaw cycles in PBS. The conditioned medium (CM) was prepared according to the following protocol. Six million trophozoites were placed in culture flasks containing 3 mL of fresh Bacto Casitone medium without FBS and incubated at 37°C for 24 h. The supernatant or CM was removed and centrifuged again at 1,500 ×g for 10 min and finally passed through a 0.22 *μ*m Durapore membrane (Millipore, Bedford, MA). Next, the samples were precipitated with absolute ethanol (J.T. Baker, USA) at a 3 : 1 ratio and stored at −20°C for 2 h, and the CM was centrifuged at 6,000 ×g for 30 min. The protein concentrations of the TCEs and CM were quantified according to the Bradford method [22]; the TCEs and CM were stored at −80°C until use.

### 2.3. Protease Inhibitors

For the inhibition assays, TCE or CM was preincubated for 1 h at 37°C with protease inhibitors with constant agitation. The inhibitor concentrations were as follows: for cysteine proteases, 10 mM* p*-hydroxymercuribenzoate (*p*HMB); for serine and cysteine proteases, 5 mM phenyl-methyl sulfonyl fluoride (PMSF); and as a specific serine protease inhibitor, 1 mM aprotinin (Sigma-Aldrich, St. Louis, MO).

### 2.4. Substrate Gel Electrophoresis

Protease activities were determined by performing electrophoresis of the TCEs and CM in 10% SDS-PAGE copolymerized with bovine hololactoferrin (h-bLf), human hololactoferrin (h-hLf), human hemoglobin (hHb), human holotransferrin (h-hTf), equine ferritin (eqF), and porcine skin gelatin (Pg) as a protease substrate. All of the substrates had a final concentration of 1 mg/mL (Sigma-Aldrich).

To determine the protein patterns of the TCEs and CM, 40 *μ*g of total protein was loaded per well. For the zymography assay, only 20 *μ*g of protein was loaded per well. As an experimental control, Bacto Casitone medium was loaded in all assays (25 *μ*L).

Electrophoresis was performed at 4°C in an ice bath and at a constant voltage (80 V) for 1 h; the gels were washed twice for 30 min with agitation in 2.5% (v/v) Triton X-100 solution (Sigma-Aldrich). The gels were then incubated overnight with 100 mM sodium acetate (pH 5.0), 100 mM Tris-HCl (pH 7.0), or 100 mM glycine (pH 9.0). All buffers contained 2 mM CaCl_2_ with or without 2 mM DTT. Finally, the gels were stained with 0.5% (w/v) Coomassie Brilliant Blue R-250 for 30 min. Protease activities were identified as clear bands on a blue background. All the assays were performed in triplicate. Inhibition analyses were performed using the ImageJ program (http://rsb.info.nih.gov/nih-image/).

## 3. Results

### 3.1. Proteases Present in Conditioned Medium and Total Crude Extracts in* N. fowleri*

**Figure 1:**
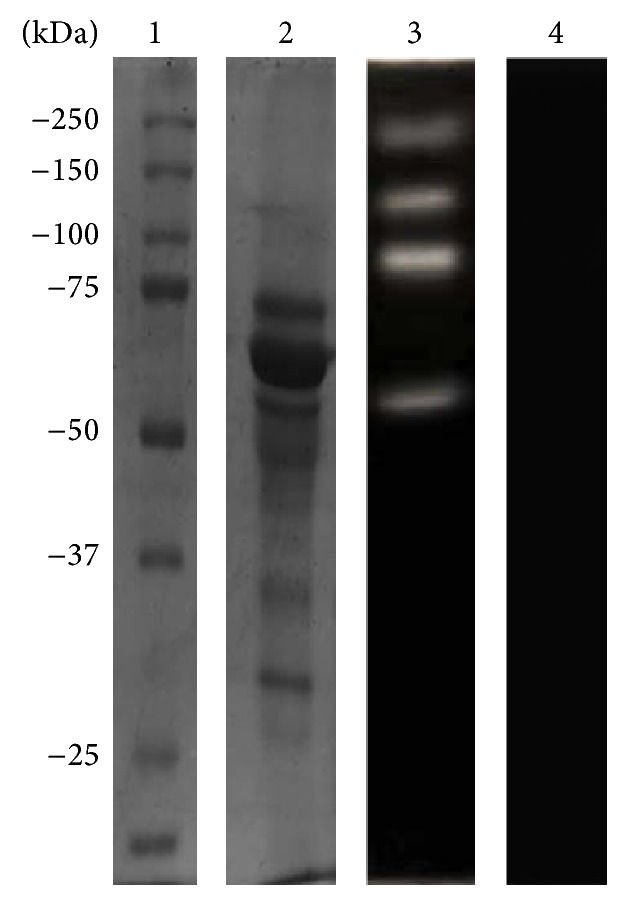
Conditioned medium proteins by SDS-PAGE and gelatin-PAGE 10%. Molecular weights (lane 1). Conditioned medium in the SDS-PAGE pattern protein (lane 2), zymogram in 0.1% porcine gelatin (lane 3). Bacto Casitone medium was loaded as a control (lane 4).

CM (Figure 1) and TCEs (see Supplementary Figure S1 in Supplementary Material available online at http://dx.doi.org/10.1155/2015/416712) were separated by 10% SDS-PAGE and stained with Coomassie Brilliant Blue. The CM protein pattern exhibited proteins between 100 and 30 kDa (Figure 1, second lane). We also evaluated the proteolytic activity of CM in 10% PAGE copolymerized with 0.1% porcine gelatin at pH 7 and 37°C. We observed four degradation bands, with calculated MWs of 172, 135, 80, and 60 kDa (Figure 1, lane 3). The Bacto Casitone medium was also analyzed as a control by 10% PAGE copolymerized with 0.1% gelatin; the medium did not produce any degradation bands (Figure 1, lane 4).

### 3.2. *N. fowleri* Proteases Can Degrade Hololactoferrin

To analyze the ability of* N. fowleri* to degrade iron-binding proteins, we performed zymography assays employing different substrates. We found that both CM and TCE (Supplementary Figures S1 and S2) were able to degrade bovine hololactoferrin (h-bLf) and human hololactoferrin (h-hLf).

**Figure 2:**
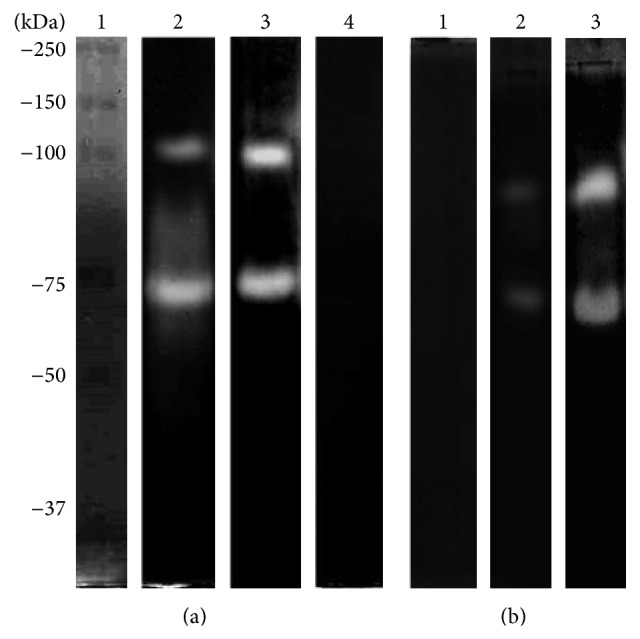
Zymography assay in 10% PAGE copolymerized with 0.1% (w/v) holo-bLf. (a) CM was evaluated at different pHs at 37°C: molecular weight marker (lane 1), pH 5 (lane 2), pH 7 (lane 3), and pH 9 (lane 4). (b) Effects of protease inhibitors evaluated at pH 7 37°C: pHMB (lane 1), PMSF (lane 2), and aprotinin (lane 3).

**Figure 3:**
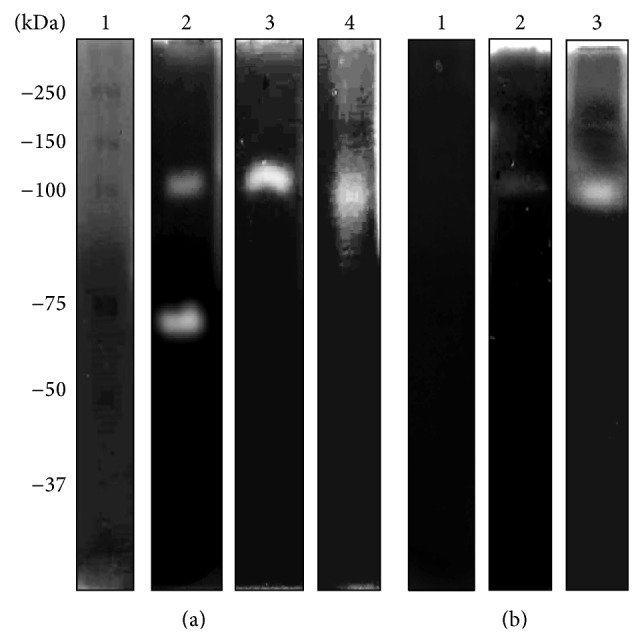
Zymography assay in 10% PAGE copolymerized with 0.1% (w/v) holo-hLf. (a) CM was evaluated at different pHs at 37°C: molecular weight marker (lane 1), pH 5 (lane 2), pH 7 (lane 3), and pH 9 (lane 4). (b) Effects of protease inhibitors evaluated at pH 7 and 37°C: pHMB (lane 1), PMSF (lane 2), and aprotinin (lane 3).

When CM was analyzed by h-bLf-PAGE at pH 5, two bands, with MWs of 100 and 75 kDa, were observed (Figure 2(a), lane 2); at pH 7, the same proteolytic pattern was found (Figure 2(a), lane 3). At pH 9, no degradation activity was detected (Figure 2(a), lane 4). In the case of h-hLf-PAGE, only two proteolytic bands (MW: 100 and 75 kDa) were found at pH 5 (Figure 3(a), lane 2) and only one band (100 kDa) was found at pH 7 (Figure 3(a), lane 3). In contrast with h-bLf-PAGE, we observed proteolytic activity at pH 9 and an MW of 100 kDa (Figure 3(a), lane 4). The activity bands of CM with all substrates are summarized in Table 1; those of TCE are given in Table 2.

Additionally, we evaluated whether the proteolytic pattern was produced by cysteine proteases. To elucidate this activity, we performed assays using specific (pHMB), partial (PMSF), and nonspecific (aprotinin) inhibitors of cysteine proteases. As anticipated, in both h-bLf and h-hLf, the degradative activity of CM at pH 7 and 37°C was abrogated only by the specific inhibitor of cysteine proteases, pHMB (Figures 2(b) and 3(b), lane 1). Moreover, we observed partial inhibition with PMSF (Figures 2(b) and 3(b), lane 2), and no effect was found with high concentrations of aprotinin (Figures 2(b) and 3(b), lane 3). Similar assays were performed for TCE, and similar results were obtained with pHMB, which inhibited all protease activities (Supplementary Figures S1 and S2).

### 3.3. Proteases Present in* N. fowleri* Degrade Human Holotransferrin (h-hTf)

**Figure 4:**
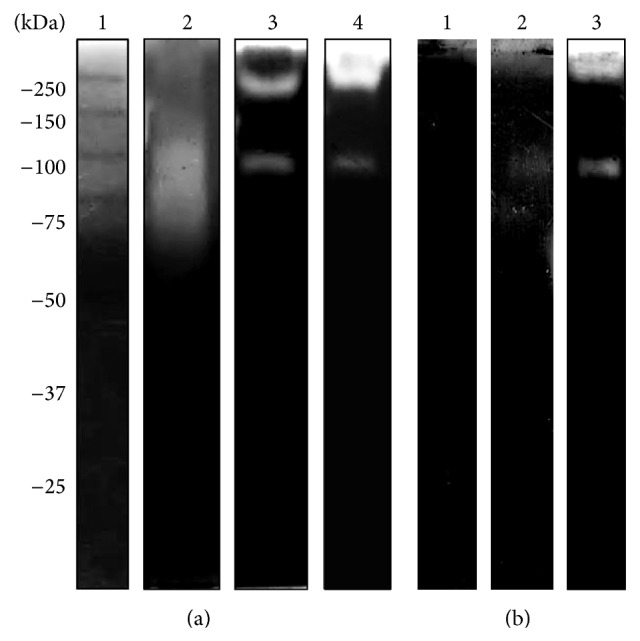
Zymography assay in 10% PAGE copolymerized with 0.1% (w/v) holo-hTf. (a) CM was evaluated at different pHs at 37°C: molecular weight marker (lane 1), pH 5 (lane 2), pH 7 (lane 3), and pH 9 (lane 4). (b) Effects of protease inhibitors evaluated at pH 7 and 37°C: pHMB (lane 1), PMSF (lane 2), and aprotinin (lane 3).

The results of the h-hTf zymography revealed activities at all the pH levels evaluated. At pH 5, we found one intense band of degradation with an MW of 100 kDa (Figure 4(a), lane 2); activation at pHs 7 and 9 revealed two activities at 180 and 100 kDa, respectively (Figure 4(a), lanes 3 and 4). In this case, we also evaluated the effect of inhibitors in CM and found a result similar to that described for hololactoferrin. Specific inhibition was achieved with cysteine protease inhibitor (pHMB) (Figure 4(b), lane 1). Similarly, we only evaluated the inhibition at a physiological pH and temperature (pH 7; 37°C). In the case of TCE activity, we detected degradation at pHs 5 and 7, and the inhibition was correlated with the results described for CM (Supplementary Figure S3).

### 3.4. *N. fowleri* Proteases Have No Effect on Ferritin

**Figure 5:**
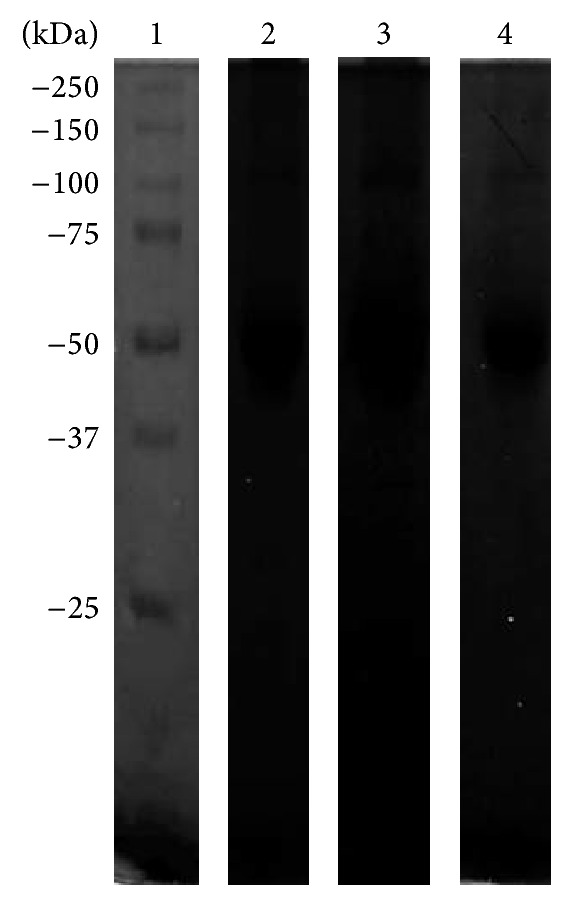
Zymography assay in 10% PAGE copolymerized with 0.1% (w/v) eqF. CM was evaluated at different pHs at 37°C: molecular weight marker (lane 1), pH 5 (lane 2), pH 7 (lane 3), and pH 9 (lane 4).

Ferritin, another iron-storing molecule, was also analyzed. This protein has been used as an indicator of iron availability in humans (liver and serum). To analyze the effect of* Naegleria* proteases on ferritin, we used equine ferritin as a substrate because this protein presents high homology with human ferritin. Interestingly, in the zymograms with ferritin as the substrate, we did not observe any degradation in CM (Figure 5) or TCE (Supplementary Figure S4).

### 3.5. *N. fowleri* Proteases Can Degrade Hemoglobin

**Figure 6:**
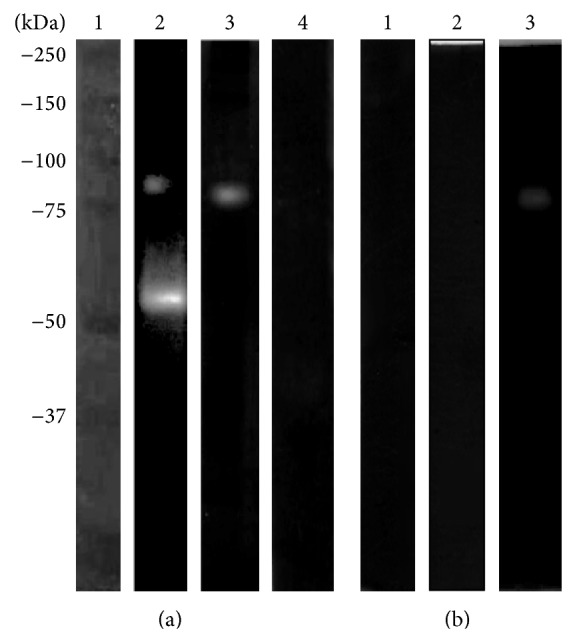
Zymography assay in 10% PAGE copolymerized with 0.1% (w/v) hHb. (a) CM was evaluated at different pHs at 37°C. Molecular weight marker (lane 1), pH 5 (lane 2), pH 7 (lane 3), and pH 9 (lane 4). (b) Effects of protease inhibitors: pHMB (lane 1), PMSF (lane 2), and aprotinin (lane 3).

Hemoglobin, a well-known iron-carrier protein that is present in erythrocytes, is another substrate possibly encountered by* N. fowleri* during its invasion process. Consequently, we also copolymerized hemoglobin with acrylamide (hHb-PAGE). The results revealed degradation activity in CM (Figure 6(a)) but not in TCE (Supplementary Figure S5). The degradation bands identified had MWs of 80 and 65 kDa at pH 5 and only one band of 80 kDa at pH 7; no activity was found at pH 9 (Figure 6(a), lanes 2–4). The main inhibition was observed with both pHMB and PMSF (Figure 6(b), lanes 1, 2) but not with aprotinin (Figure 6(b), lane 3).

## 4. Discussion

In nature, the distribution and proliferation of* N. fowleri* have been associated with the proportion of Fe^2+^ in the vertical distribution of the water column [23]. However, during the process of invading the nasal cavity,* N. fowleri* encounters an iron-deficient environment and an extracellular glycoprotein of the innate immune response (where the ferric iron is chelated by apolactoferrin) [24, 25]. Additionally, the deficiency of iron in* N. fowleri* cultures has been shown to be responsible for reduced proliferation of the parasite [26]. Furthermore, the addition of molecules that contain iron (e.g., Hb, hemin, and protoporphyrin IX) provides resistance to lysis by complement, increases motility, and induces proliferation in culture [27–29]. However, the mechanism by which* N. fowleri* obtains iron during PAM has not been described.

In the case of the parasitic protozoan* E. histolytica,* several mechanisms (such as receptors and proteases) are used to obtain iron [30]. In this context, we evaluated the ability of* N. fowleri* to degrade iron-binding proteins including hololactoferrin, hemoglobin, holotransferrin, and ferritin. The zymography assays revealed a degradation of the specific substrates; moreover, this strategy allowed us to estimate the molecular weight of the proteases involved in this process.

We observed proteolytic activity in gels copolymerized with both holo-hLf and holo-bLf; this is the first report of* N. fowleri* to demonstrate that this free-living amoeba degrades these proteins. Interestingly, we observed one activity band of 100 kDa conserved in human and bovine holo-Lf. In contrast, the presence of a 75 kDa activity band was predominant in bovine holo-Lf. This slight difference might be due to the homology of human and bovine Lf (69%) [31, 32]. In basal conditions, apolactoferrin is produced by serous cells in the nasal cavity (ratio 200–300 *μ*g/mL) [33]; many reports confirm that the apo-Lf concentration increases during microbial infection or tissue damage [34, 35]. The main role of apo-Lf is its iron-chelating activity, and when this occurs, both globular domains of apo-Lf are occupied by iron ions (holo-Lf or h-Lf) [35]. It has recently been reported that* E. histolytica* can ingest human h-Lf; moreover, this protein is transported in acid vesicles. Additionally, the researchers performed PAGE copolymerized with h-hLf to elucidate the type of proteases present in these vesicles and found that total crude extracts of* E. histolytica* contained cysteine proteases; in contrast with our results, they did not observe activity in supernatants [20]. In the case of* N. fowleri,* we identified activity in both CM and TCEs; additionally, when we used ethanol to precipitate CM of* E. histolytica*, we observed bands of degradation (data not shown). These results support the hypothesis that* N. fowleri* can obtain iron by degrading h-hLf. The different patterns of CM degradation of h-hLf and h-bLf may be supported by the difference of homology in the sequence of amino acids in these proteins. The differential degradation by nonsecreted proteases suggests internalization and processing of hLf; unfortunately, little is known about endocytosis and vesicular trafficking in* N. fowleri*. Therefore, it is important to study the effect of h-hLf in* N. fowleri* and also to elucidate the mechanisms of uptake of hHb, h-Tf, and ferritin, which are other important sources of iron in the human body. Human transferrin is mainly synthesized by the liver, although other tissues and organs, such as the brain, are also able to produce transferrin [36, 37]. Both ferritin and transferrin are found in oligodendrocytes; these cells are present in the optic nerve and in both the gray and white matter of the cerebral cortex, cerebellum, and olfactory bulbs. Astrocytes and glia cells are also positive for ferritin, transferrin, and iron [36, 38, 39]. These brain cells have been described to interact with* N. fowleri* during the later stages of PAM [7, 40]. Therefore, we evaluated whether* N. fowleri* could also degrade these iron-binding proteins, and we observed degradation of holo-hTf by CM and TCE, but these extracts had no effect on ferritin.

The effect of cysteine proteases in ferritin has been demonstrated in bacteria [41], fungi [42], and some protozoa [30, 43]. The differences between CM and TCE may depend on a specific process (endocytosis) or on degradation caused by a wide range of proteases [44]. In the present work, the nondegradation of ferritin was surprising because it is the main iron storage protein in the human body; furthermore, we expected that* N. fowleri* proteases would degrade this protein because* N. gruberi* has been reported to bind ferritin on the cell surface [45]. However, it is possible that coupling ferritin in* N. fowleri* only occurs in a transitory event that culminates in capping formation [2, 46]. Another possible mechanism involved in nondegradation is the high molecular weight of the protein [47].

The hemorrhagic and lytic zones in the brain during PAM progression include the presence of erythrocytes. The phagocytosis of red blood cells has been reported in* N. fowleri* [48, 49]. In erythrocytes, the iron is stored in a ferrous oxide form in the hemoglobin. Certain bacteria, such as* Pseudomonas aeruginosa*,* Micrococcus luteus*,* Serratia marcescens,* and* Staphylococcus aureus*, have proteases that degrade Hb [15, 50, 51]. In* S. aureus*, it was reported that, during nasal colonization, invasion depends on the Hb concentration [50]. However, in* E. histolytica*, many studies have investigated erythrophagocytosis and the vesicular process involved in erythrocyte degradation. The proteolysis of red blood cells also includes the participation of cysteine proteases [18, 30, 52, 53]. Recently, in* N. fowleri*, the activity of secretion/excretion proteases against Hb was determined [12]. However, in our study, we evaluated the activity of CM and TCEs under physiological conditions (pH 7 at 37°C) and determined the MW of these hemoglobin proteases.

It is important to mention that in the zymography assays we evaluated the type of proteases involved in the degradation of each substrate evaluated. For this, we employed a cysteine proteases inhibitor, pHMB, serine and partially cysteine protease inhibitor, PMSF, and serine inhibitor, aprotinin [54, 55]. The results showed in all the substrates that the inhibition of proteases activities was mainly with pHMB; these data suggest that* N. fowleri* cysteine proteases are involved in the degradation of iron-binding proteins. Additionally, we evaluated the effect of the conventional E-64 over* N. fowleri* cysteine proteases to discriminate the possible family or clan of proteases [55, 56]. The results with E-64 inhibitor in CM revealed a partial inhibition in the case of holo-bLf and holo-hTf but total inhibition of h-hLf and hHb. In TCE only partial inhibition was observed in holo-bLf and total inhibition for holo-hLf and holo-hTf (data not shown). The results of inhibition suggest that family C1 (cathepsin-like) or clan CA and clan CD could be involved in the iron-binding proteins degradation [56]. However, it is important to mention that this hypothesis needs to be elucidated and it is subject of another study. Finally, it is important to mention that our research was focused mainly on secretion proteases involved in the invasive process. Thus, it will be important to perform new experiments using TCEs to evaluate the intracellular pathways involved in the degradation process of iron-binding proteins. This could be correlated with the different patterns observed in the CM and TCEs.

## 5. Conclusion

The present results support the hypothesis that* N. fowleri* can obtain iron during the initial and final stages of PAM. Our findings indicate that the proteases involved in this process vary over a wide range of MWs, from 50 to 150 kDa. Additionally, we hypothesize that* N. fowleri* depends on iron to survive and proliferate during PAM.

## Supplementary Material

The results of the supplementary figures showed the degradation activities of total crude extract (TCEs) of N. fowleri. We analyzed the degradation pattern of different iron-binding proteins such as human and bovine holo-lactoferrin, human holo-transferrin, equine ferritin, and human hemoglobin at different pHs and at 37°C. Also we evaluated the type of proteases using different proteases inhibitors.

## Figures and Tables

**Table 1:** Conditioned medium (CM). Locations of bands present in the zymography assays using different substrates: (X) proteases at pH 7; (∗) proteases that appear at pH 5 and 9.

MW		Substrate															
		Pg	h-bLf			h-hLf			h-hTf			eqF			hHb		
	pH	7	5	7	9	5	7	9	5	7	9	5	7	9	5	7	9
180										X	∗						
172		X															
135		X															
100			∗	X		∗	X	∗	∗	X	∗						
80		X													∗	X	
75			∗	X		∗											
65															∗		
60		X															

**Table 2:** Total crude extract (TCE). Locations of bands present in the zymography assays using different substrates: (X) proteases at pH 7; (∗) proteases that appear at pH 5 and 9.

MW		Substrate															
		Pg	h-bLf			h-hLf			h-hTf			eqF			hHb		
	pH	7	5	7	9	5	7	9	5	7	9	5	7	9	5	7	9
150		X	∗	X					∗								
130		X			∗												
100		X	∗	X	∗	∗	X			X							
70		X	∗	X													
75									∗								
60		X							∗	X							
50						∗											
