# Supplementary material for: Iron-Binding Protein Degradation by Cysteine Proteases of Naegleria fowleri
Source: Biomed Res Int. 2015 May 18;2015:416712. doi: 10.1155/2015/416712 (PMC4450812; doi:10.1155/2015/416712)
Supplement: Supplementary file 1 — The results of the supplementary figures showed the degradation activities of total crude extract (TCEs) of N. fowleri. We analyzed the degradation pattern of different iron-binding proteins such as human and bovine holo-lactoferrin, human holo-transferrin, equine ferritin, and human hemoglobin at different pHs and at 37°C. Also we evaluated the type of proteases using different proteases inhibitors. [file 416712.f1.pdf]

Supplementary figures

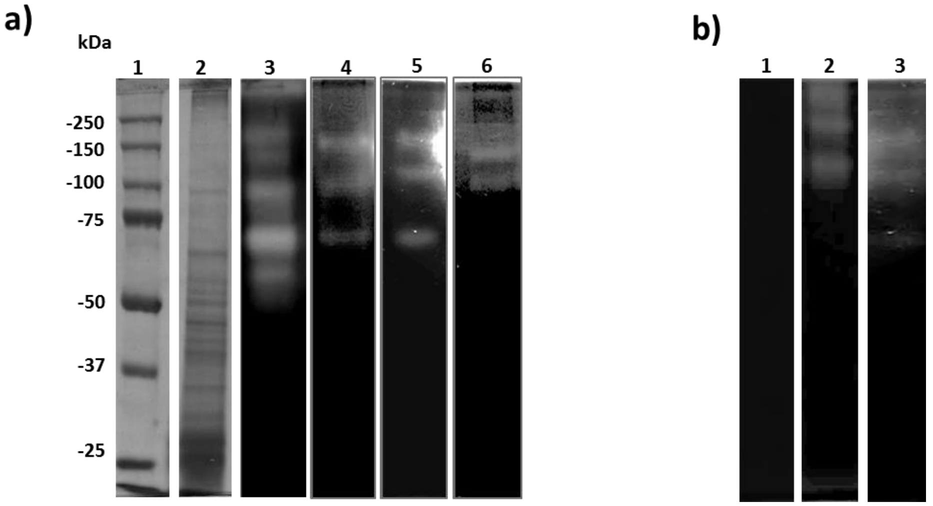

S1. Bovine holo-lactoferrin. (a) *N. fowleri* TCE evaluated at different pHs. Molecular weight marker (lane 1), *N. fowleri* TCE protein pattern in SDS-PAGE (lane 2), 10% PAGE co-polymerized with 0.1% porcine gelatin pH 7 (lane 3); 10% PAGE co-polymerized with bovine hLf TCE evaluated at different pHs at 37°C: pH 5 (lane 4), pH 7 (lane 5), pH 9 (lane 6). (b) Effects of protease inhibitors at pH 7 at 37°C: pHMB (lane 1), PMSF (lane 2), aprotinin (lane 3).

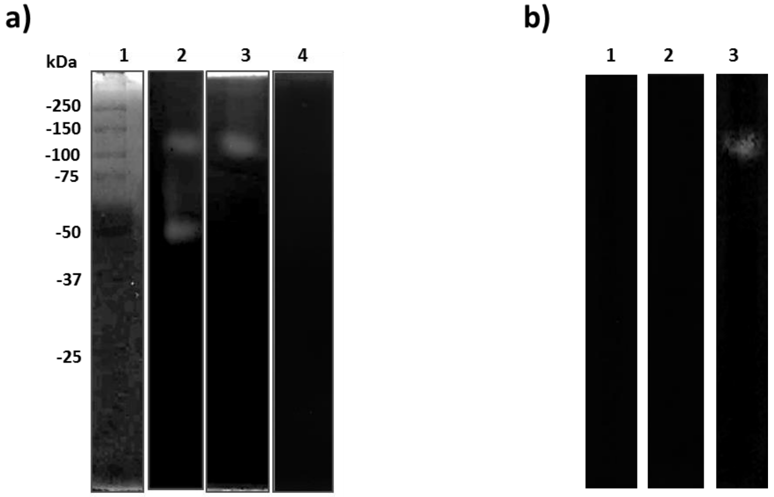

S2. Zymography assay in 10% PAGE co-polymerized with 0.1% (w/v) holo-hLf. (a) TCEs were evaluated at different pHs at 37°C: pH 5 (lane 2), pH 7 (lane 3), pH 9 (lane 4). (b) Effects of protease inhibitors at pH 7 at 37°C: pHMB (lane 1), PMSF (lane 2), aprotinin (lane 3).

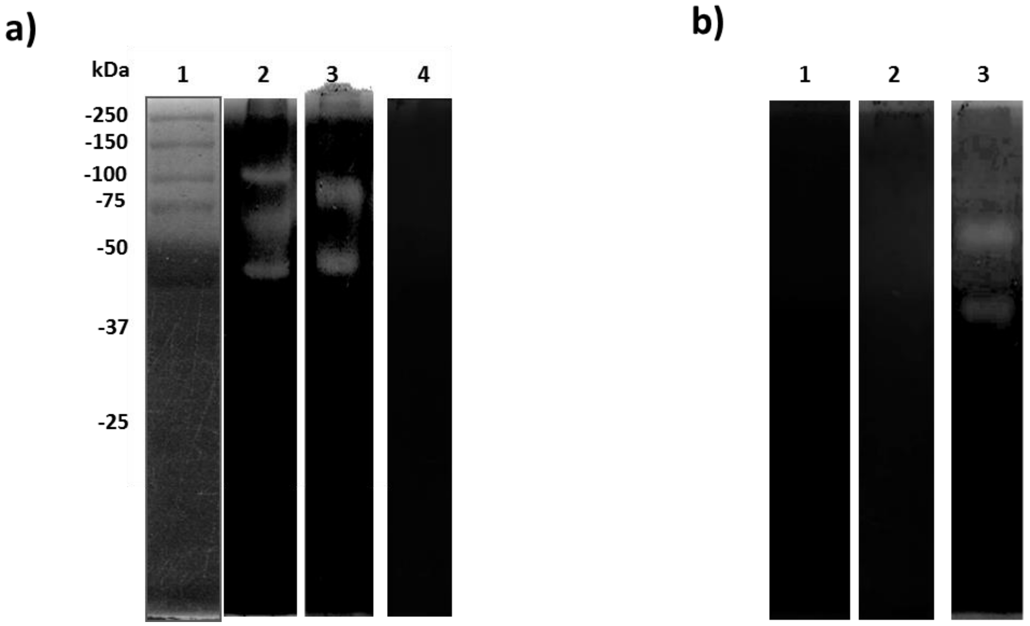

S3. Zymography assay in 10% PAGE co-polymerized with 0.1% (w/v) holo-hTf. (a) TCEs were evaluated at different pHs at 37°C: pH 5 (lane 2), pH 7 (lane 3), pH 9 (lane 4). (b) Effects of protease inhibitors at pH 7 at 37°C: pHMB (lane 1), PMSF (lane 2), aprotinin (lane 3).

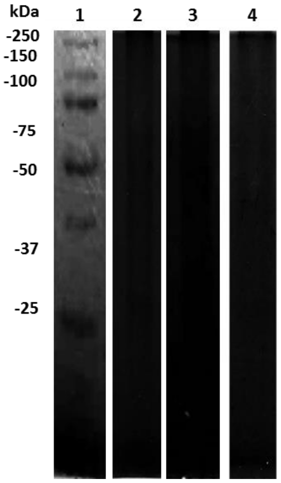

S4. Zymography assay in 10% PAGE co-polymerized with 0.1% (w/v) eqF. TCEs were evaluated at different pHs at 37°C: pH 5 (lane 2), pH 7 (lane 3), pH 9 (lane 4).

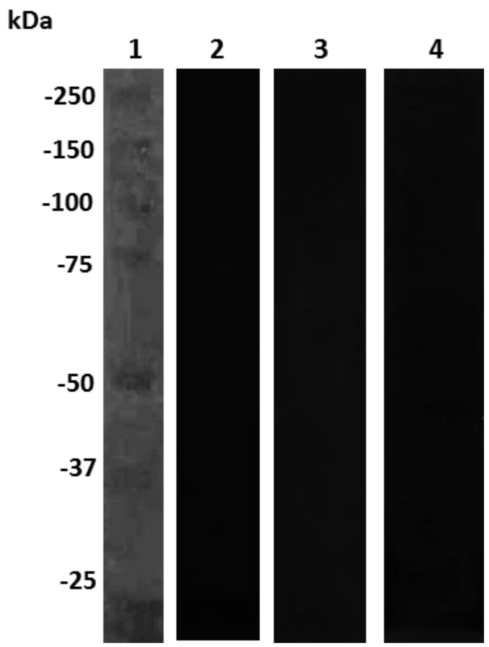

S5. Zymography assay in 10% PAGE co-polymerized with 0.1% (w/v) hHb. TCEs were evaluated at different pHs at 37°C: pH 5 (lane 2), pH 7 (lane 3), pH 9 (lane 4).
